# Multilocus Variable-Number-Tandem-Repeats Analysis (MLVA) distinguishes a clonal complex of *Clavibacter michiganensis* subsp*. michiganensis* strains isolated from recent outbreaks of bacterial wilt and canker in Belgium

**DOI:** 10.1186/1471-2180-13-126

**Published:** 2013-06-05

**Authors:** Joanna Zaluga, Pieter Stragier, Johan Van Vaerenbergh, Martine Maes, Paul De Vos

**Affiliations:** 1 Laboratory of Microbiology, Department of Biochemistry and Microbiology, Ghent University, K.L. Ledeganckstraat 35, Gent, B-9000, Belgium; 2 Plant-Crop Protection, Institute for Agricultural and Fisheries Research, ILVO, Burg. Van Gansberghelaan 96, Merelbeke, B-9820, Belgium; 3 BCCM/LMG Bacteria collection - Laboratory of Microbiology Department of Biochemistry and Microbiology, Ghent University, K.L. Ledeganckstraat 35, Gent, B-9000, Belgium

**Keywords:** *Clavibacter*, MLVA, Bacterial typing, Epidemiology, Bacterial wilt and canker, Plant pathogen

## Abstract

**Background:**

*Clavibacter michiganensis* subsp*. michiganensis* (Cmm) causes bacterial wilt and canker in tomato. Cmm is present nearly in all European countries. During the last three years several local outbreaks were detected in Belgium. The lack of a convenient high-resolution strain-typing method has hampered the study of the routes of transmission of Cmm and epidemiology in tomato cultivation*.* In this study the genetic relatedness among a worldwide collection of Cmm strains and their relatives was approached by *gyrB* and *dnaA* gene sequencing*.* Further, we developed and applied a multilocus variable number of tandem repeats analysis (MLVA) scheme to discriminate among Cmm strains.

**Results:**

A phylogenetic analysis of *gyrB* and *dnaA* gene sequences of 56 Cmm strains demonstrated that Belgian Cmm strains from recent outbreaks of 2010–2012 form a genetically uniform group within the Cmm clade, and Cmm is phylogenetically distinct from other *Clavibacter* subspecies and from non-pathogenic *Clavibacter*-like strains. MLVA conducted with eight minisatellite loci detected 25 haplotypes within Cmm. All strains from Belgian outbreaks, isolated between 2010 and 2012, together with two French strains from 2010 seem to form one monomorphic group. Regardless of the isolation year, location or tomato cultivar, Belgian strains from recent outbreaks belonged to the same haplotype. On the contrary, strains from diverse geographical locations or isolated over longer periods of time formed mostly singletons.

**Conclusions:**

We hypothesise that the introduction might have originated from one lot of seeds or contaminated tomato seedlings that was the source of the outbreak in 2010 and that these Cmm strains persisted and induced infection in 2011 and 2012. Our results demonstrate that MLVA is a promising typing technique for a local surveillance and outbreaks investigation in epidemiological studies of Cmm*.*

## Background

*Clavibacter michiganensis* subsp*. michiganensis,* a Gram positive bacterium, is the causative agent of bacterial canker and wilting, one of the most destructive bacterial diseases in tomato [1]. Contaminated tomato seeds are considered to be the main source of infection. The bacterium survives for a long period of time in seeds, soil and plant debris [2,3]. Every year, new or reoccurring outbreaks are detected causing substantial economic losses worldwide [4]. Bacterial canker was described for the first time in 1905 in Michigan, USA, and since that moment it has been reported in nearly all tomato growing areas of the world [3]. Difficulties in controlling the spread of the pathogen, the lack of resistant tomato varieties and severity of disease symptoms led to the classification of Cmm as quarantine organisms. Cmm is listed as an A2 quarantine pest by the European and Mediterranean Plant Protection Organization (EPPO) [2] in Europe and in many countries all over the world [1].

The epidemiology and the population structure of Cmm in areas where outbreaks of Cmm are common remains scantily investigated and poorly understood. Recent studies describing outbreaks of Cmm in Europe and Asia [5-8] have shed some light on this issue. In Italy a clonal population of Cmm was responsible for the outbreak in 2007 [9]. A high homogeneity was also observed among strains isolated from 2002 to 2007 in Canary Islands suggesting a single introduction of the pathogen as a source of infection [6]. Primary infections in many countries were attributed to the introductions of contaminated tomato seeds and/or seedlings [7,10]. These findings indicate that seeds play an important role in long-distance spread of the pathogen. A direct link between tomato cultivar, year or place of isolation and Cmm type mostly could not be recognized [6,8,9] except the outbreak in 2001 in Turkey where bacterial canker was detected only on one tomato cultivar ‘Target’ [11]. Interestingly, in Israel and Serbia Cmm strains showing the same haplotypes were repeatedly isolated from the same locations during several subsequent years [7,10]. Reoccurring outbreaks suggest that despite intensified efforts for eradication, reliable control of this disease remains an unattainable goal. The limited progress in improving its management is mainly due to the sporadic nature of the disease outbreaks and to limited and scattered epidemiological data. Therefore, access to an accurate, efficient and cost-effective strain typing technique could be very useful.

Bacterial typing techniques are applied to quickly and reliably differentiate closely related strains in an epidemiological survey, to determinate the relatedness among the strains and to track their origin and pathways of spread. Over the past decades a variety of different typing methods have been developed to generate strain-specific patterns. They are also applied for comprehensive investigation of bacterial population structure and dynamics. A range of methods has already been applied to study the diversity of *Clavibacter,* particularly to investigate Cmm strains. Rep-PCR (repetitive-element-based PCR), a relatively easy and fast technique, was shown to be of moderate utility [8], mainly because of the lack of a database and the rather low discriminatory power needed to study closely related strains. Moreover, rep-PCR is mostly not portable between different laboratories [12]. PFGE (pulsed-field gel electrophoresis of macro-restricted bacterial DNA), one of the oldest techniques used in epidemiology, is labor intensive and expensive but is still used as a gold standard in typing of some bacterial species [10,13]. PFGE was applied to study the diversity of Cmm strains from outbreaks in Serbia [7] and in Israel [10] where the results of PFGE showed similar resolution of those obtained by gene sequence analysis and rep-PCR, respectively. Also, AFLP, a high resolution molecular typing method was applied by De Leon and coworkers to study genetic diversity of Cmm strains from Canary Islands [6]. This technique generated more bands per strain and resulted in more reproducible and robust discriminatory clustering of the strains [6]. Highly reproducible multilocus sequence typing (MLST) was used to analyze Cmm population from Serbia. Cmm strains were divided into seven groups and the results were confirmed by PFGE analysis [7].

MLVA (Multiple-Locus Variable number tandem repeat Analysis) is a PCR-based typing technique that has been widely applied in medical microbiology [14]. It takes advantage of the inherent variability encountered in regions with a number of tandem repeats. The origin of the repetitive regions can be accounted to slipped strand mispairing events occurring during DNA duplication, in which repetitive regions are incorrectly copied resulting in deletion or insertion of one or several copies of the repeat [15]. PCR primers designed to board different VNTR (Variable Number of Tandem Repeats) regions in the genome can be easily combined in a multiplex PCR in an MLVA scheme. The differences between strains are assessed by the different lengths of the repeats visualized by gel electrophoresis or automated fragment analysis on a sequencer. From these sizes, the number of repeat units at each locus can be deduced. The resulting information forms a strain-specific numerical code which can be easily compared to a reference database. The MLVA technique was introduced to bacterial typing as a promising alternative or a complement to already existing typing methods such as AFLP, MLST, rep-PCR or PFGE. The discriminatory power of MLVA is generally higher than other standard typing techniques [16]. However, the final result is group dependent and can vary considerably between different bacterial species. VNTRs have been used to discriminate among individual strains within many food-borne pathogens with little genetic differences, including *Escherichia coli* O157:H7 [17] and *Vibrio cholerae*[18] and to study other important human pathogens, such as *Neisseria gonorrhoeae*[19], *Streptococcus pneumoniae*[20], and *Mycobacterium tuberculosis*[21]. MLVA has been extensively used for tracking transmissions of important human and animal pathogens [22,23] and for typing monomorphic bacterial pathogens including *Bacillus anthracis*[24] and *Yersinia pestis*[25]. To date, several MLVA schemes have been published on plant pathogens such as *Xanthomonas citri* pv. *citri*[31], *X. oryzae* pv*. oryzicola*[26]*, Pseudomonas syringae* pv. *maculicola* and *tomato*[27]*, Xylella fastidiosa*[28] and on fungi e.g. *Aspergillus flavus*[29], but not for *Clavibacter* subspecies. In plant pathogens, such as *Xanthomonas arbolicola* pv*. pruni,* MLVA was proposed as a complementary molecular typing method to AFLP, BOX and ERIC-PCR [30]. In the epidemiological study of pathotypes of *Xanthomonas citri* MLVA was compared to AFLP and insertion sequence ligation-mediated PCR (IS-LM-PCR) and was found the best method to describe the variations among strains originating from the same country or group of neighboring countries [31].

The objectives of this study were: 1) to characterize a Belgian population of Cmm strains by a newly developed MLVA scheme; 2) to compare its genetic variability with some strains of Cmm isolated in other countries; 3) to investigate whether the strains responsible for bacterial canker outbreaks in Belgium in 2010–2012 have one or several infection sources and 4) to assess the genetic relatedness of the Cmm strains from Belgium by *gyrB* and *dnaA* gene sequence analysis.

## Methods

### Bacterial strains

The bacterial strains used in this study are listed in Table 1. The strains were obtained from the BCCM/LMG Bacteria Collection (Ghent, Belgium), the GBBC (ILVO Plant Clinic, Merelbeke, Belgium) and the PD collection (Wageningen, The Netherlands). The *Clavibacter* strain subset consisted of five type strains Cmm LMG 7333^T^ (species type strain), *Clavibacter michiganensis* subsp. *nebraskensis* (Cmn) LMG 5627^T^, *Clavibacter michiganensis* subsp. *sepedonicus* (Cms) LMG 2889^T^, *Clavibacter michiganensis* subsp. *insidiosus* (Cmi) LMG 3663^T^, *Clavibacter michiganensis* subsp. *tessellarius* (Cmt) LMG 7294^T^, two non-pathogenic *Clavibacter*-like strains and fifty five Cmm originating from Belgian outbreaks and other geographical locations. Twenty three Cmm strains were sampled from symptomatic tomato plants in fields and greenhouses in northeast Belgium. They were isolated from five different tomato cultivars and seven different locations, in the period February 2010 till February 2012 (Table 1). *Clavibacter*-like isolates from tomato seed are phenotypically similar to Cmm in the common diagnostic semi-selective media and are identified as Cmm in the standard tests but are non-pathogenic to tomato [32,33]. They were isolated according to the current method for detection of Cmm in tomato seed recommended by International Seed Federation (ISF) [34]. The strains were cultured aerobically on MTNA (mannitol, trimethoprim, nalidixic acid, amphotericin) medium without antibiotics [35] at 25°C for 24-48 h. Stock cultures were stored at −80°C in Microbank^TM^ beads (Pro-Lab Diagnostics, Canada).

**Table 1:** ***Clavibacter *strains included in the study**

**Nr**	**Strain nr **^**1**^	**Name**^**2**^	**Host of isolation**	**Cultivar**	**Geographical**^**3 **^**origin**	**Year of isolation**	**Alternative number**	**MLVA group**^**4**^
1	GBBC 283	Cmm	*Solanum lycopersicum*	*-*	Belgium	2007	-	G
2	GBBC 1082*	Cmm	*Solanum lycopersicum*	*Admiro*	Belgium	2011	-	W
3	GBBC 1083*	Cmm	*Solanum lycopersicum*	*Admiro*	Belgium	2011	-	W
4	GBBC 1086*	Cmm	*Solanum lycopersicum*	*-*	Belgium	2011	-	W
5	GBBC 1389	Cmm	*Solanum lycopersicum*	*-*	Belgium	2012	-	W
6	GBBC 297*	Cmm	*Solanum lycopersicum*	*Growdena*	Belgium (Berlaar)	2010	-	W
7	GBBC 310*	Cmm	*Solanum lycopersicum*	*Growdena*	Belgium (Berlaar)	2010	-	W
8	GBBC 298*	Cmm	*Solanum lycopersicum*	*Admiro*	Belgium (Beveren)	2010	-	W
9	PD 5734	Cmm	*Solanum lycopersicum*	*Adelaide*	Belgium (Duffel)	1998	GBBC 178 = LMG 26621	E
10	GBBC 296*	Cmm	*Solanum lycopersicum*	*Growdena*	Belgium (Duffel)	2010	-	W
11	GBBC 311*	Cmm	*Solanum lycopersicum*	*Growdena*	Belgium (Duffel)	2010	-	W
12	GBBC 316*	Cmm	*Solanum lycopersicum*	*Growdena*	Belgium (Duffel)	2010	-	W
13	GBBC 1060*	Cmm	*Solanum lycopersicum*	*Admiro*	Belgium (Duffel)	2010	-	W
14	GBBC 282	Cmm	*Solanum lycopersicum*	*Plaisance*	Belgium (Geel)	2007	PD 5741 = LMG 26626	U
15	GBBC 285	Cmm	*Solanum lycopersicum*	*Admiro*	Belgium (Kontich)	2008	PD 5742 = LMG 26627	I
16	PD 1953	Cmm	*Solanum lycopersicum*	*Dombito*	Belgium (Melsele)	1990	GBBC 100 = LMG 26622	G
17	GBBC 1604*	Cmm	*Solanum lycopersicum*	*Growdena*	Belgium (Melsele)	2010	-	W
18	GBBC 301*	Cmm	*Solanum lycopersicum*	*Growdena*	Belgium (Melsele), ng	2010	-	W
19	GBBC 300*	Cmm	*Solanum lycopersicum*	*Growdena*	Belgium (Melsele), og	2010	-	W
20	GBBC 1064*	Cmm	*Solanum lycopersicum*	*Growdena*	Belgium (Putte)	2010	-	W
21	GBBC 1606*	Cmm	*Solanum lycopersicum*	*Levanzo*	Belgium (Rijkevorsel)	2010	-	W
22	PD 5737	Cmm	*Solanum lycopersicum*	*Concreto 622*	Belgium (Rumst)	1984	GBBC 103 = LMG 26624	J
23	PD 5733	Cmm	*Solanum lycopersicum*	*Durinta*	Belgium (Rumst)	1996	GBBC 150 = LMG 26620	L
24	GBBC 1609*	Cmm	*Solanum lycopersicum*	*DRW 7749*	Belgium (Rumst)	2010	-	W
25	PD 5736	Cmm	*Solanum lycopersicum*	*Rianto*	Belgium (St-Katelijne-Waver)	1983	GBBC 101 = LMG 26623	I
26	GBBC 312*	Cmm	*Solanum lycopersicum*	*Growdena*	Belgium (Waver)	2010	-	W
27	GBBC 1061*	Cmm	*Solanum lycopersicum*	*Growdena*	Belgium (Waver)	2010	-	W
28	GBBC 1605*	Cmm	*Solanum lycopersicum*	*Bigdena*	Belgium (Waver)	2010	-	W
29	GBBC 303*	Cmm	*Solanum lycopersicum*	*DRW 7749*	Belgium (Wervic)	2010	-	W
30	GBBC 304*	Cmm	*Solanum lycopersicum*	*Bigdena*	Belgium (Wervic)	2010	-	W
31	GBBC 308*	Cmm	*Solanum lycopersicum*	*DRW 7749*	Belgium (Wervic)	2010	-	W
32	PD 5753	Cmm	*Solanum lycopersicum*	*-*	Algeria	1985	CFBP 2495	Q
33	LMG 5644	Cmm	*Solanum lycopersicum*	*-*	Canada	1982	-	O
34	Cl01TF02^#^	Cmm	*Solanum lycopersicum*	*-*	Canary Islands (Tenerife)	2003	-	C
35	GBBC 1077	Cmm	*Solanum lycopersicum*	*-*	France	2010	-	W
36	GBBC 1078	Cmm	*Solanum lycopersicum*	*-*	France	2010	-	W
37	GBBC 1079	Cmm	*Solanum lycopersicum*	*-*	France	2010	-	P
38	GBBC 1080	Cmm	*Solanum lycopersicum*	*-*	France	2010	-	P
39	PD 5721	Cmm	*Solanum lycopersicum*	*-*	France	2006	LMG 26819	S
40	PD 5719	Cmm	*Solanum lycopersicum*	*-*	France	2008	-	K
41	PD 5749	Cmm	*Solanum lycopersicum*	*Admiro*	France	2007	GBBC 261 = LMG 26628	A
42	PD 4545	Cmm	*Solanum lycopersicum*	*-*	Germany	2003	LMG 26617	D
43	LMG 7333^T^	Cmm	*Solanum lycopersicum*	*-*	Hungary	1957	-	R
44	PD 1386	Cmm	*Solanum lycopersicum*	*-*	Italy	1961	NCPPB 1064 = LMG 3687	K
45	GBBC 242	Cmm	*Solanum lycopersicum*	*Daniëlla*	Morocco	2003	PD 5750 = LMG 26629	M
46	LMG 5602	Cmm	*Cyphomandra betacea*	*-*	New Zealand	1967	-	X
47	PD 5699	Cmm	*Solanum lycopersicum*	*-*	Portugal	1998	-	N
48	LMG 3695	Cmm	*Solanum lycopersicum*	*-*	Romania	1970	-	F
49	PD 4149	Cmm	*Solanum lycopersicum*	*-*	Slovenia	2001	LMG 26619	B
50	ES 2686.1^#^	Cmm	*Solanum lycopersicum*	*-*	Spain (Granada)	2002	-	J
51	PD 1664	Cmm	*Solanum lycopersicum*	*-*	Sweden	-	LMG 26805	R
52	PD 5722	Cmm	*Solanum lycopersicum*	*-*	Switzerland	2007	-	T
53	PD 1948	Cmm	*Solanum lycopersicum (seeds)*	*-*	Taiwan	1988	PD 1683 = LMG 26625	G
54	GBBC 247	Cmm	*Solanum lycopersicum*	*-*	The Netherlands (Velden)	2004	-	H
55	LMG 3681	Cmm	*Solanum lycopersicum*	*-*	United Kingdom	1956	NCPPB 382	V
56	PD 5751	Cmm	*Solanum lycopersicum*	*-*	USA	1998	GBBC 172 = LMG 26630	O
57	LMG 26807	*Clavibacter*-like	*Solanum lycopersicum (seeds)*	*-*	India	2000	PD 5683	na
58	LMG 26810	*Clavibacter*-like	*Solanum lycopersicum (seeds)*	*-*	Chile	2007	PD 5686	na
59	LMG 3663^T^	Cmi	*Medicago sativa*	*-*	USA	1955	NCPPB 1109	na
60	LMG 5627^T^	Cmn	*Zea mays*	*-*	USA	1971	NCPPB 2581 = LMG 3700^T^	na
61	LMG 7294^T^	Cmt	*Triticum aestivum (Aestivum Group)*	*-*	-	1978	ATCC 33566	na
62	LMG 2889^T^	Cms	*Solanum tuberosum*	*-*	Canada	1968	NCPPB 2137	na

^1^Bacterial collection abbreviations: LMG-BCCM/LMG-Bacterial Collection (Ghent, Belgium), PD-PD collection (Wageningen, The Netherlands) and GBBC-GBBC collection (ILVO, Merelbeke, Belgium).

*Nursery BPK, Duffel, Belgium, ^*#*^ Instituto Canario de Investigaciones Agrarias (Tenerife, Spain), eight underlined strains were included in an initial testing of VNTR loci.

^2^Cmm-*Clavibacter michiganensis* subsp. *michiganensis*, Cmn-*Clavibacter michiganensis* subsp. *nebraskensis*, Cms-*Clavibacter michiganensis* subsp. *sepedonicus*, Cmi-*Clavibacter michiganensis* subsp. *insidiosus*, Cmt-*Clavibacter michiganensis* subsp. *tessellarius*.

^-^Cultivar unknown; geographical origin unknown; year of isolation unknown; no alternative number.

^3^ng-new greenhouse, og-old greenhouse.

^4^na-not applicable.

### DNA extraction, amplification and sequencing

Total genomic DNA was extracted according to the guanidium-thiocyanate-EDTA-sarkosyl method described by Pitcher et al. [36] which was adapted for Gram-positive bacteria by a pre-treatment with lysozyme (5 mg/μl lysozyme in TE buffer). Amplification and sequencing primers are listed in Table 2. The expected amplicons were generated with the Qiagen Taq DNA polymerase kit (supplemented with a Q-Solution) and GeneAmp® dNTP’s (Applied Biosystems, Belgium) according to the manufacturer specifications and with primers from Sigma Aldrich (Belgium). Amplicons were purified using the Nucleofast®96 PCR clean up membrane system (Macherey-Nagel, Germany). Sequencing PCR was performed in a total volume of 10 μl with 3 μl of a purified amplicon, 0.286 μl of BigDye^™^ mixture (Terminator Cycle Sequencing Kit version 3.1, Applied Biosystems), 1x sequencing buffer and 1.2 μM of each of the amplification primers listed in Table 2. The PCR program consisted of 30 cycles (96°C for 15 s, 35°C for 1 s, 60°C for 4 min). Subsequently, the sequencing products were purified using the BigDye XTerminator Kit (Applied Biosystems) and analyzed on a 3130xl Genetic Analyzer (Applied Biosystems).

**Table 2:** Primers sequences used in this study

**Nr**	**Name**	**Amplification primers (seq)**	**Sequencing primers**	**Position in a genome **^**a**^	**Gene (ORF)**	**Product size range (bp)**
1	Clav-VNTR2	F-5′-GGTCTACGTCGACGAGGTCTT-3′	F- 5′-GCACCGCCACATGGAGAG-3′	3107999-3108175	putative zinc-dependant oxidoreductase (putative carboxylesterase)	165-300
		F-5′-TTCGCGTTCCTCACCAAC-3′	R-5′-GTCGACGCGCTACGGGAG-3′			
2	Clav-VNTR5	F-5′-GGGCCCGATCAACGACAT-3′	F-5′-CGGACACGTCAGCCTACC-3′	2130562-2130864	putative transcriptional regulator (MerR family)	200-475
		F-5′-CATCGAGTCGGCCCTGGT-3′	F-5′-GAGATCGCCACGCAGCTC-3′			
3	Clav-VNTR9	F-5′-GCACGGCGTCACGGTCAG-3′	F-5′-CGAGGAGTGGAACCAGGCCG-3′	2183702-2183742	putative arylesterase (putative transcriptional regulator, LysR-family)	150-200
		F-5′-AGCTCGCGAAGCCGTCCAC-3′	F-5′-CGAAGGCCTCCAAGGGCCAG-3′			
4	Clav-VNTR13	F-5′-GTCGTGGTGCGGGGTCGT-3′	F-5′-ACGTCCAGCATTCCTCCA-3′	468356-468428	putative NAD(FAD)-dependent dehydrogenase	200-250
		F-5′-TGACCGGCACGTCAAGGAGA-3′	F-5′-ACGTCCAGCATTCCTCCA-3′			
5	Clav-VNTR15	F-5′-GCCGTCTCTGCGTCTTTC-3′	F-5′-CCTCGAGATGACACCTGAAT-3′	2684839-2684928	putative duplicated acetyltransferase	130-200
		F-5′-ATGAGACGTCCAGCAGTGG-3′	F-5′-GATGTGTACGATCCGCTCTC-3′			
6	Clav-VNTR16	F-5′-GTCGCCTACGAGTTCATGGT-3′	F-5′-GTCACGGCGCCCTAGGAACC-3′	1929615-1929835	putative glycine/betaine ABC transporter (Putative DNA or RNA helicase)	175-300
		F-5′-AGCTCCTCAACAGCCTCGT-3′	F-5′-TCGGCCAGTGCAGCGTCA-3′			
7	Clav-VNTR22	F-5′-ACACCCGCCCGACTAGACC-3′	F-5′-GACAGGCCGGTCGGAGGAAT-3′	549526-549594	putative two-component system response regulator	175-225
		F-5′-CGGAAGCTGCACGACGAC-3′	F-5′-GTGCGCGGCGTCGGATAC-3′			
8	Clav-VNTR26	F-5′-CCTTCGCGGTGCGGATCA-3′	F-5′-GACGAGGACGGTGTCGAG-3′	178774-178838	putative urea amidolyase (conserved hypothetical protein)	150-175
		F-5′-GGGATCGTCGACGGCATGAG-3′	F-5′-GCTGGTGATCGTCTCCAACT-3′			
9	*gyrB 2 F*	F-5′-ACCGTCGAGTTCGACTACGA-3′	The same as amplification primers	6588-7113	DNA gyrase, subunit B	525
	*gyrB 4R*	F-5′- CCTCGGTGTTGCCSARCTT-3′				
10	*dnaA F*	5-TACGGCTTCGACACCTTCG-3	The same as amplification primers	412-1345	replication initiation factor (RIF)	933
	*dnaA R*	5-CGGTGATCTTCTTGTTGGCG-3				

^a^genome of Cmm NCPPB 382 (AM711867).

### Sequence analysis

In the frame of the European project QBOL (Quarantine Barcoding Of Life) we developed a *gyrB* barcode that was proven suitable to identify members of the genus *Clavibacter* at the subspecies level (http://www.q-bank.eu/) [32]. Moreover, *gyrB* gene was used in MLST schemes developed to type Cmm strains [7,33,37]. *DnaA* sequence was shown a good taxonomic marker to identify and classify plant pathogenic bacteria such as *Clavibacter, Xanthomonas* and *Ralstonia*[38]. The partial sequencing of *dnaA* was successfully used to study genetic diversity of non-pathogenic *Clavibacter*-like strains and to identify members of the genus *Clavibacter* (J. Zaluga, data unpublished). The *gyrB* and *dnaA* sequences were assembled with BioNumerics version 5.1 (Applied Maths, Belgium) and aligned using ClustalW [39]. *GyrB* sequences and *dnaA* sequences were checked by amino acid translation with Transseq (http://www.ebi.ac.uk/Tools/emboss/transeq/) and presence of the GyrB and DnaA protein domain was confirmed with BlastP [40]. *DnaA* and *gyrB* amplicons were 675 bp and 440 bp long (equal length was used for all strains), respectively. A phylogenetic tree was constructed on *dnaA*-*gyrB* concatenated sequence data with Molecular Evolutionary Genetics Analysis software (Mega 5.1) [41], using the Maximum Likelihood method with the Tamura-Nei model [42] and 1000 bootstrap replicates. The position of the sequenced *gyrB* and *dnaA* amplicons were checked by comparison to the reference Cmm genome sequence (AM711867). Newly generated *gyrB* and *dnaA* sequences have following accession numbers KC521547-521623 and have been deposited in NCBI database. Each unique sequence of a gene was assigned an allele number and the combination of allele numbers for each isolate defined the haplotype. Number of haplotypes, haplotype diversity and number of polymorphic sites were estimated for *gyrB* and *dnaA* genes using DnaSP version 5.0 [43]. Percentages of polymorphic sites at the analyzed loci were calculated by dividing the number of polymorphic positions by the total length of the gene. The Discriminatory Power (D) was calculated using a discriminatory power calculator (http://insilico.ehu.es/mini_tools/discriminatory_power/index.php). The Discriminatory Power (D), as shown by Hunter can be expressed by the formula of Simpson’s index of diversity, which reads:

$$D=1-\frac{1}{N\left(N-1\right)}\sum_{j=1}^{s}x_{j}\left(x_{j}-1\right)$$

Where D is the index of discriminatory power, N the number of unrelated strains tested, S the number of different types, and xj the number of strains belonging to the jth type, assuming that strains will be classified into mutually exclusive categories. Thus, a D value of 1.0 would indicate that a typing method was able to distinguish each member of a strain population from all other members of that population. Conversely, an index of 0.0 would indicate that all members of a strain population were of an identical type. An index of 0.50 would mean that if one strain was chosen at random from a strain population, then there would be a 50% probability that the next strain chosen at random would be indistinguishable from the first [44].

### Design of VNTR primers

The complete genome sequence of *Clavibacter michiganensis* subsp*. michiganensis* NCPPB 382 deposited under accession number AM711867 was screened for VNTR loci. Tandem Repeat Finder program (http://tandem.bu.edu) [45] was used to detect potential VNTR loci. Primer3 software [46] was used to design locus-specific amplifications and sequencing primers in regions flanking VNTR loci. Eight loci (Table 3) of 20 bp to 45 bp long tandem repeat (TR) units were selected. TRs longer than 20 bp were chosen to enable easier interpretation of results from an agarose gel. Primer pairs targeting single locus alleles were manually designed in the conserved regions to obtain amplicons of no more than 450 bp in length.

**Table 3:** **Range of repeats, size of repeats, numbers of alleles and diversity indices (Simpson’s, Hunter-Gaston and Shannon-Wiener) for each VNTR locus used to investigate 56 *Clavibacter michiganensis *subsp*. michiganensis *strains**

**Locus**	**Range of repeats**	**Size of repeat (bp)**	**Nr of alleles**	**Simpson’s diversity index**^**a**^	**Hunter-Gaston diversity index**^**a**^	**Shannon-Wiener index of diversity**^**b**^
Cmm-V5	3-8.5	46	6	0.652	0.664	1.3377
Cmm-V9	1-3	20	3	0.577	0.588	0.932
Cmm-V13	1-3	35	3	0.534	0.544	0.8225
Cmm-V2	2-5	45	3	0.53	0.54	0.844
Cmm-V26	1-2	33	2	0.494	0.503	0.677
Cmm-V15	3-5	34	3	0.417	0.425	0.7334
Cmm-V16	2-6.5	47	5	0.392	0.399	0.8864
Cmm-V22	1-3	26	2	0.504	0.514	0.5811

Diversity Index (for VNTR data) = A measure of the variation of the number of repeats at each locus. Ranges from 0.0 (no diversity) to 1.0 (complete diversity).

^a^Calculated by V-DICE (http://www.hpa-bioinformatics.org.uk/cgi-bin/DICI/DICI.pl).

^b^Calculated in BioNumerics v 5.1.

### VNTR PCR amplification and sequencing

The PCR mixture had a total volume of 25 μl, containing 1 x PCR buffer (100 mM Tris–HCl, 15 mM MgCl_2_, 500 mM KCl [pH 8.3]) (Qiagen), dNTP’s 0.2 mM each, 0.6 μM of each primer, 0.5 U DNA Taq polymerase, and 50–60 ng template DNA. The PCR amplifications were performed under following conditions: 3 min denaturation step at 94˚C; 35 cycles of 94˚C for 1 min, annealing at 60˚C for 1 min, and extention at 72˚C for 1 min; and a final extension step at 72˚C for 10 min. Amplified products were run on a 2.5% Gel Pilot® Small Fragment Agarose (Qiagen) at 110 V for 2.5 hrs at 4°C using 25 bp size marker (Invitrogen), and visualized by ethidium bromide staining. PCR amplicons from one representative strain per different locus of a particular VNTR were sequenced using sequencing primers (Table 2) according to the sequencing protocol described above for *gyrB* and *dnaA* genes.

### VNTR analysis and statistics

Product sizes were estimated and the exact number of repeats present was calculated using a derived allele-naming table, based on the number of repeats which could theoretically be present in a PCR product of a given size, allowing for extra flanking nucleotides and primer size. Theoretical number of repeats was confirmed subsequently by sequencing. Loci were named simply on the basis of the order in which they were found by the initial search. VNTR allele calls were analyzed in BioNumerics as ‘character’ data. Composite datasets were created for the eight Clav-VNTR loci. Distance trees were derived by clustering with the unweighted pair group method with arithmetic means (UPGMA), using ‘categorical’ character table values. All markers were given equal weight, irrespective of the number of repeats. The percentages in the dendrogram reflect the percentage of homology between the specific markers. Relatedness between the different haplotypes was investigated based on comparison of allelic profiles using the minimum spanning tree (MST) method from BioNumerics v 5.1. We used the classical criterium of one allelic mismatch to group haplotypes into clonal complexes. In order to assess the evolutionary relatedness between haplotypes the MLVA data was analyzed taking into account the number of repeat differences. The type strain LMG 7333^T^ served as a reference and a starting point for calculations of the differences in other strains. For each VNTR locus the Hunter–Gaston and Simpson’s diversity indices were calculated using the VNTR diversity and confidence extractor software (V-DICE) available at the Health Protection Agency bioinformatics tools website (http://www.hpa-bioinformatics.org.uk/cgi-bin/DICI/DICI.pl) [47]. Shannon-Wiener index of diversity was calculated using BioNumerics version 5.1.

## Results

### Assessment of genetic diversity among *Clavibacter* strains

**Figure 1:**
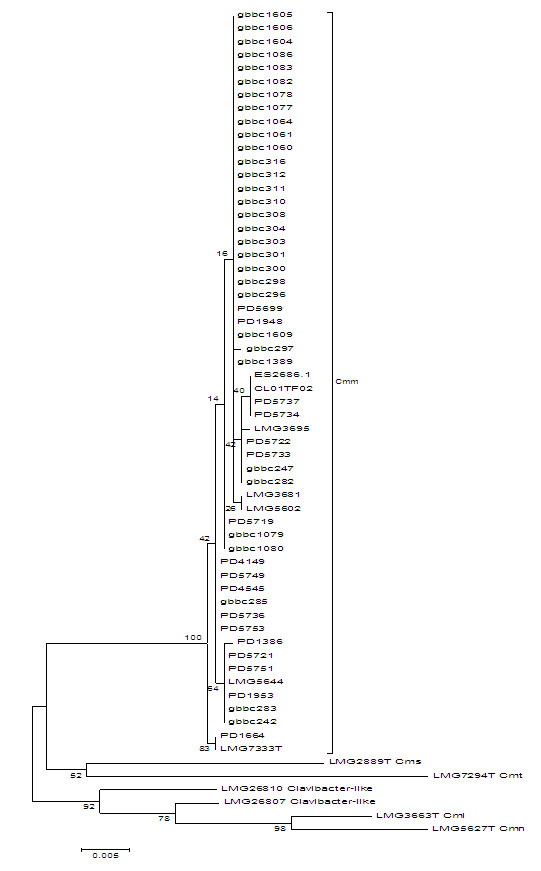
**Phylogenetic analysis of concatenated tree of *dnaA *and *gyrB *sequences based on 1115 bp.** Maximum Likelihood (ML) tree with the Tamura-Nei model of 62 *Clavibacter* strains with bootstrap values generated from 1000 replicates.

In total, 62 strains representing the *Clavibacter* subspecies and non-pathogenic *Clavibacter-*like strains were included in this study. The identity of included Cmm strains was confirmed by analysis of the *gyrB* and *dnaA* gene sequences*.* The gene sequence analyses were performed on several related *Clavibacter* strains in order to study the genetic diversity in the genus *Clavibacter*. Phylogenetic analysis of two tested genes confirmed a clear separation of *Clavibacter* subspecies and a distinct position of non-pathogenic *Clavibacter-*like strains. Phylogenetic relationship between the *Clavibacter* subspecies and non-pathogenic *Clavibacter-*like strains was strongly supported by high bootstrap values (Figure 1). The number of polymorphic sites was 47 (10.7%) and 87 (12.9%), for *gyrB* and *dnaA*, respectively. It has to be noted that diversity among Cmm strains, especially among strains from recent Belgian outbreaks, was small which resulted in a limited number of clusters. Despite a low genetic diversity, a number of groups could be distinguished in a Cmm cluster (Figure 1). The largest cluster, containing Belgian strains from recent outbreaks and two French strains from 2010 (GBBC 1077 and GBBC 1078), was separated from the Cmm strains isolated previously in Belgium (Figure 1). Furthermore, strains originating from the same location mostly grouped together, such as French strains GBBC 1079, GBBC 1080 and PD 5719. However, based on the concatenated Maximum Likelihood tree of *gyrB* and *dnaA* no clear geographical separation among Cmm strains could be demonstrated. In *gyrB* and *dnaA* trees (data not shown) and in a concatenated tree *Clavibacter* subspecies are separated from each other and from non-pathogenic strains which suggests that they present the same phylogenetic information (Figure 1).

### Development and implementation of MLVA

**Figure 2:**
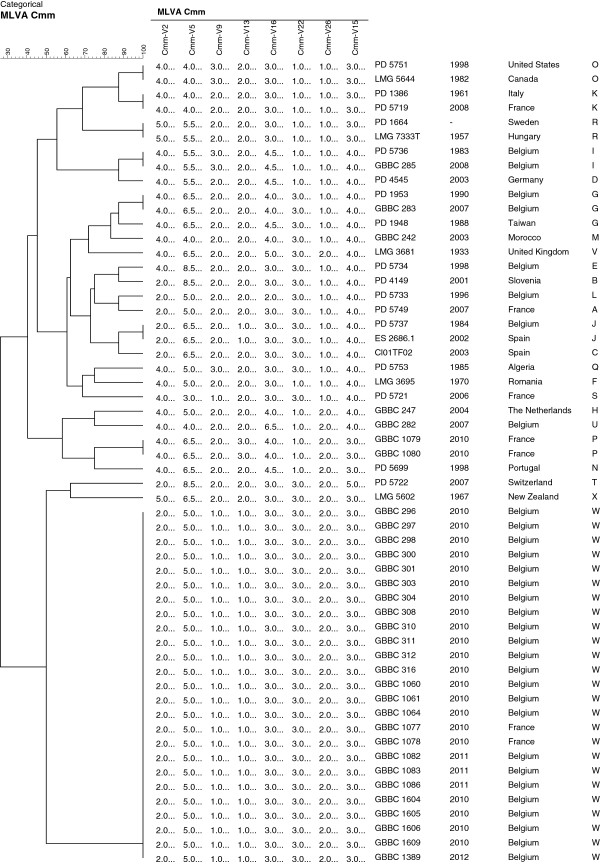
**Grouping of 56 Cmm strains using categorical values and the UPGMA (Unweighted-Pair Group Method with Arithmetic Mean) algorithm, generated with BioNumerics 5.1 software.** Numbers in the Cmm-V2-26 columns indicate repeat counts.

In parallel with the sequence analysis Cmm strains were investigated with MLVA. Fifty eight VNTR loci were identified in the genome of Cmm NCPPB 382. Thirty one of them were tested on a set of eight genetically diverse Cmm strains originating from geographically spread locations (Table 1). Subsequently, eight loci that were successfully amplified and showed to be polymorphic in the tested subset of strains were selected for further analysis. Successful amplification was obtained in all tested Cmm strains. Regarding the non-pathogenic, seed-borne *Clavibacter-*like strains the results varied from no amplification for Clav-VNTR5 or unspecific (more than one band, not expected product size) bands in Clav-VNTR26 (data not shown). Similar findings were observed for *Clavibacter* subspecies other than Cmm. In the cluster analysis, a total of 24 MLVA types were detected among 56 Cmm strains when the data from eight loci were combined, with allele numbers per locus ranging from two (Clav-VNTR22, Clav-VNTR26) to six (Clav-VNTR5) (Table 3, Figure 2). A large cluster, comprised of Cmm strains from recent Belgian outbreaks together with two French strains isolated in 2010, exhibited identical MLVA haplotypes. Strains from other countries formed mostly a separate branch or a cluster with two strains with an identical MLVA haplotype. No direct connection between strains from recent Belgian outbreaks of 2010–2012 and other Belgian strains included in this study could be observed. Remarkably, Belgian strains PD 5736 and GBBC 285, isolated in 1983 and 2008, respectively, showed the same MLVA haplotypes. In the concatenated tree of *gyrB* and *dnaA* these two Belgian strains clustered together among strains originating from other countries (Figure 1). Similar findings were observed for other two Belgian strains PD 1953 and GBBC 283, isolated in 1984 and 2002, respectively.

**Figure 3:**
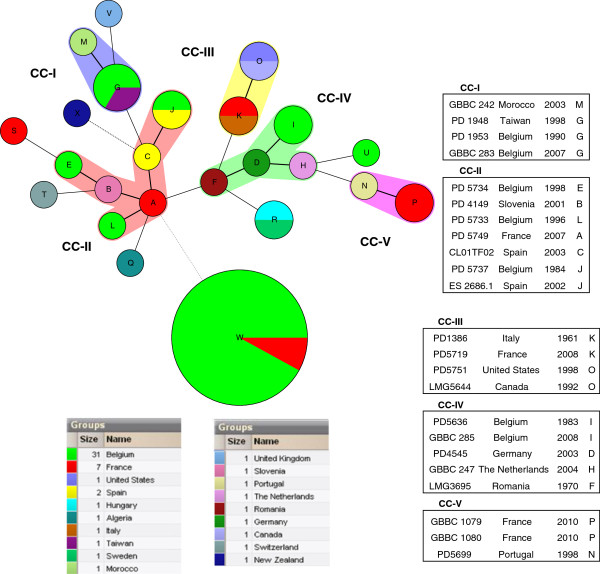
**Minimum spanning tree of 56 Cmm strains based on eight VNTR loci.** Each circle represents an MLVA type with a size corresponding to the number of strains that share an identical MLVA type. MLVA types connected by a thick solid line differ from one another by one VNTR locus, while MLVA types connected by a thin solid line differ by two VNTR loci. MLVA types that differ from each other by three, four or more VNTR loci are connected by dashed and dotted lines. MLVA types were distinguished to define clonal complexes and to group in zones MLVA types that differ from one another by at most two locus variants. Letters visible on each circle are corresponding to strains described in Table 1. CC-Clonal complex.

The discriminatory abilities of the MLVA technique was determined by calculating the discriminatory index (*D*) for 56 typed strains. MLVA differentiated 25 Cmm strains and showed a level of discrimination, with a *D* value of 0.8006. The discriminatory power of each VNTR was estimated by the number of alleles detected and the allele diversity. The number of different alleles ranged from two for Cmm-V22 and Cmm-V26 to six for Cmm-V5. Highest allelic diversities measured by Hunter–Gaston, Simpson’s and Shannon-Wiener diversity indices were 0.664; 0.652; 1.3377, respectively and were observed for the loci Clav-VNTR5 (Table 3). For the set under study, 27 different alleles of eight VNTR loci were observed. The relationship among the strains based on MLVA results is presented in a minimum spanning tree (MST) (Figure 3). The 56 Cmm strains were resolved into 24 types distributed into five complexes separating double locus variants (DLV). In addition, a large clonal group of Belgian strains from recent outbreaks (W), six singletons (S, T, Q, X, V, U) each represented by an isolate from a different country, and one separate group consisting of two strains (R) were detected (Table 1, Figure 3). Based on MLVA results, strains from Belgian outbreaks 2010–2012 were identical; no differences could be observed between strains originating from different years of isolation, tomato varieties or geographic locations in Belgium (Table 1, Figure 2, and Figure 3). To receive more information about evolutionary relatedness of strains from Belgium and France the MLVA data was analyzed taking into account the number of repeat differences (Additional file 1: Figure S1). Interestingly, Belgian strain PD 5737 and French strain PD 5749 clustered closer to ES2686.1 and CL01TF02 strains isolated in Spain during bacterial canker outbreak in 2002–2003. Moreover, these four strains showed to have a more similar MLVA haplotype to the group of strains from recent Belgian outbreaks 2010–2012.

## Discussion and conclusion

Over the last few decades, bacterial canker has been frequently detected in tomato production areas, leading to substantial financial and economical losses. Only during the last three years several local outbreaks of Cmm were reported in Belgium. In some cases, reoccurring infections were detected in the primarily contaminated farms, suggesting a persistence of an initial infection source. Despite a quite frequent detection of tomato canker and wilting in Belgian tomato production areas there is little known about the genetic diversity of Cmm strains which hinders the correct conclusions about the probable sources of epidemics and transmission routes of Cmm.

This study is the first MLVA approach developed for efficient genotyping of Cmm strains. To date typing of Cmm strains was performed by RAPD-PCR [6], BOX-PCR [8,48], AFLP [6], PFGE [10] and MLST [7]. Despite the fact that some of these methods were found to have a good resolution most of them have limitations such as a poor interlaboratory portability or limited exchangeability of results that were generated on a specific machine or compared to an in-house database. Nowadays, fully sequenced genomes give a unique opportunity for a development of more robust and accurate typing methods such as MLVA. Its advantages, such as, high reproducibility, exchangeability of results and the possibility to add loci greatly facilitates epidemiological studies of economically important pathogens such as Cmm.

In this work, Clav-VNTR5 showed to be the most polymorphic loci with five different alleles and the highest HGDI of 0.664. Combined data from MLVA analysis of all eight investigated loci resulted in 25 different haplotypes and a discriminatory power of 0.8006. Cmm strains from the recent epidemics in Belgium in 2010–2012 showed identical MLVA haplotypes which suggests that a clonal population was responsible for these outbreaks. The presence of the same MLVA haplotypes of Cmm strains from 2011 and 2012 could mean that bacteria persisted in the used equipment, devices or soil and induced the outbreaks in the following years. Population of Belgian strains isolated from 2010–2011 is epidemiologically related to at least two French strains that exhibited the same MLVA haplotype. Moreover, based on minimum spanning tree, Belgian strains were found to be evolutionary related to the French strain PD 5749. When MLVA data was analyzed taking into account differences in the number of repeats it appeared that two French and two Spanish strains were found to have a similar MLVA haplotype to the group of Belgian strains from 2010–2012 suggesting that there might be a common origin of these strains (Additional file 1: Figure S1). It is worth mentioning that the strain ES 2686.1 isolated in Spain in 2002 was linked to outbreaks of Cmm in 2002–2007 in Canary Islands [6]. Two French strains isolated in 2010 showed the same MLVA haplotype as strains from recent Belgian outbreaks which may imply that the contaminated material was spread also in France. Different MLVA patterns between strains from the recent Belgian outbreaks of 2010–2012 and Belgian strains isolated previously support our hypothesis about a novel introduction, presumably originating from a single lot of seeds or contaminated tomato seedlings. Remarkably, all Belgian Cmm strains from 2010–2012 (Table 1), were purchased from the same nursery.

In this study, VNTR loci were chosen to be longer than or equal to 20 bp to simplify the interpretation of the results from an agarose gel and to allow performing the analysis in standard laboratories not equipped in sophisticated tools (fragment analyzer or sequencer) required to analyze small (a few nucleotides) differences in an amplicon size. Shorter repeats are represented in a higher number of copies and are more likely to be polymorphic [49]. However, many studies showed successful application of longer repeats which gave satisfactory resolution and discriminatory power [16,50]. Moreover, *in silico* analysis of tandem repeats in the Cmm genome NCPPB 382 revealed only a few short repeats (6–8 bp) that had remarkably higher number of copies (around 10 copies).These microsatellite loci might be investigated in the future and combined with currently available MLVA scheme. MLVA can provide phylogenetic information even with a limited number of loci [51]. MLVA assays are relatively robust [17,52] but as any other technique they have their limitations. In MLVA, a need to develop a new set of loci for every species or serovar under investigation might be necessary. Moreover, some loci are ‘not stable’ and can ‘disappear’ from some strains or lineages what will result in an uninformative ‘zero’ allele [53].

VNTRs might possibly contribute to the genomic polymorphism and/or evolution. Comparative genomics of pathogenic *Mycobacterium tuberculosis* showed that a variation in size and number of repeats, located in coding regions, can result in a variable expression of surface-exposed proteins that play a role in pathogenicity [54]. These changes could possibly help the pathogen to avoid the host immune response. Expansion or reduction of the number of tandem repeats can influence the expression, structure and activity of cellular proteins. Tandem repeats located within regulatory regions can result in a modification of gene expression at the transcriptional level [55]. All tested Clav-VNTR loci were found in putative coding regions (Table 2). At least two of them were found within genes linked to processes taking place in a cell envelope (Clav-VNTR-13: putative NAD (FAD)-dependent dehydrogenase and Clav-VNTR 16: putative glycine/betaine ABC transporter). We could speculate that variability observed within these regions might possibly help bacteria to alternate the proteins of a cell envelope. However, more research has to be performed on the role of tandem repeat copy, and virulence in Cmm.

The genetic structure of the studied strains was assessed by the sequence analysis of two housekeeping genes, *gyrB* and *dnaA,* which were previously reported to be good molecular markers for studying populations of the genus *Clavibacter*[32,38]. The phylogenetic position of Cmm strains was supported by high bootstrap values in a Maximum Likelihood tree. High similarity of Belgian strains from recent outbreaks was detected both, in a gene sequence analysis and by an MLVA typing method, supporting the hypothesis about their monomorphic nature. The percentages of polymorphic sites observed for the concatenated set of *gyrB* and *dnaA* genes (Table 4) was higher than the value obtained from five concatenated genes described in a recently published MLSA scheme of *Clavibacter michiganensis* subsp. *michiganensis,* (12 versus 8.8) [33]. Based on these parameters the genes selected in this work can be applied in MLST studies to investigate highly similar Cmm populations.

**Table 4:** **Discrimination indices for *Clavibacter *typing methods**

**Typing technique**	**Hunter-Gaston diversity index**	**Number of haplotypes**^**b**^	**Number of polymorphic sites**^**b**^	**Number of sites**	**% of polymorphic sites**
*gyrB*	0.586^b^	10	47	440	10.7
*dnaA*	0.662^b^	12	87	675	12.9
Concatenated *gyrB-dnaA*	0.758^b^	17	134	1115	12.0
MLVA	0.800^a^	25	na	na	na

^a^Calculated in discriminatory Power Calculator (http://insilico.ehu.es/mini_tools/discriminatory_power/) based on 56 Cmm strains.

^b^Calculated in DnaSP v.5 [44] based on 56 Cmm strains.

na- not applicable.

In this study, MLVA was successfully applied to investigate a genetic relationship of Cmm strains from recent Belgian outbreaks. Its discriminatory power, measured by HGDI, was higher than these of each of the tested genes, *gyrB* and *dnaA* (Table 4). Our study has shown that MLVA analysis offers better discrimination of Cmm strains (HGDI = 0.8) than the typing method based on the concatenated tree of *gyrB* and *dnaA* (HGDI = 0.758) (Table 4). A significant advantage of the MLVA method is the excellent interlaboratory reproducibility [56] which makes this method well-suited for accurate and reproducible bacterial typing applicable in epidemiological studies of *Clavibacter.* MLVA, with its high discriminatory power to separate closely related strains, might be very useful for tracking sources of epidemic outbreaks as well as for investigating various haplotypes occurring during these outbreaks, as illustrated in the differentiation of Cmm strains. The technique is fast (results within one day), easy to perform, user-friendly, cost-effective compared to other typing techniques (e.g. AFLP) with an excellent reproducibility (intra- and interlaboratory). Additionally, data storage, comparison and exchange of the results are possible and easy. Moreover, the use of fluorescence-labeled primers enables multiplex PCR and subsequent analysis in a fragment analyzer. It is worth mentioning that the MLVA scheme, derived from in silico analysis of a complete genome sequence of Cmm, was experimentally confirmed to be accurate. It is consistent with previous findings demonstrated for *Xanthomonas citri* pv*. citri* and is advantageous over other experimentally tested techniques such as AFLP or IS-LM-PCR, where *in vitro vs. in silico* accuracy values of 75% and 87%, respectively, were reported [31].

The MLVA method, with eight novel VNTR loci identified within the genome of Cmm, demonstrated its applicability as a new tool for the molecular investigation of bacterial wilting and canker outbreaks.

In the future, additional VNTR loci and C*lavibacter* isolates might enable unraveling intrapopulation genetic variation and assessing the robustness of the method for investigating bacterial canker outbreaks on a global scale.

## Competing interests

The other authors declare that they have no competing interests.

## Authors’ contributions

PS, MM, JVV and PDV conceived the study and participated in its design and coordination. JVV and PDV provided the bacterial culture collection for the study. JZ participated in the design of the study, carried out the molecular work, performed the data analysis and drafted the manuscript. PS coordinated the work and performed the statistical analysis. All authors read and approved the final manuscript.

## Supplementary Material

Additional file 1: Figure S1 Grouping of 56 Cmm strains using categorical values and the UPGMA (Unweighted-Pair Group Method with Arithmetic Mean) algorithm, generated with BioNumerics 5.1 software based on the number of repeats differences. Numbers in the Cmm-V2-26 columns indicate numbers of repeats differences. Click here for file
