# Supplementary material for: Multilocus Variable-Number-Tandem-Repeats Analysis (MLVA) distinguishes a clonal complex of Clavibacter michiganensis subsp. michiganensis strains isolated from recent outbreaks of bacterial wilt and canker in Belgium
Source: BMC Microbiol. 2013 Jun 5;13:126. doi: 10.1186/1471-2180-13-126 (PMC3691591; doi:10.1186/1471-2180-13-126)
Supplement: Additional file 1: Figure S1 — Grouping of 56 Cmm strains using categorical values and the UPGMA (Unweighted-Pair Group Method with Arithmetic Mean) algorithm, generated with BioNumerics 5.1 software based on the number of repeats differences. Numbers in the Cmm-V2-26 columns indicate numbers of repeats differences. [file 1471-2180-13-126-S1.docx]

Figure S1. Grouping of 56 Cmm strains using categorical values and the UPGMA (Unweighted-Pair Group Method with Arithmetic Mean) algorithm, generated with BioNumerics 5.1 software based on the number of repeats differences. Numbers in the Cmm-V2-26 columns indicate numbers of repeats differences.
